# Incident prescriptions for common cardiovascular medications: comparison of recent versus pre-2020 medication adherence and discontinuation in three universal health care systems

**DOI:** 10.1186/s12872-025-04492-3

**Published:** 2025-02-05

**Authors:** Candace D. McNaughton, Peter C. Austin, Cynthia A. Jackevicius, Anna Chu, Jessalyn K. Holodinsky, Michael D. Hill, Colleen M. Norris, Mukesh Kumar, Noreen Kamal, Douglas S. Lee, Nadia Khan, Manav V. Vyas, Raed A. Joundi, Moira K. Kapral, Amy Y. X. Yu

**Affiliations:** 1 ICES, Toronto, ON Canada; 2 Sunnybrook Health Sciences Centre, Sunnybrook Research Institute, University of Toronto, Room V1 39, 2075 Bayview Ave, Toronto, ON Canada; 3 Department of Medicine, University of Toronto, Toronto, ON Canada; 4 Institute of Health Policy, Management and Evaluation, University of Toronto, Toronto, ON Canada; 5 Western University of Health Sciences, Pomona, CA USA; 6 Departments of Emergency Medicine, Cumming School of Medicine, University of Calgary, Calgary, AB Canada; 7 Department of Community Health Sciences, Cumming School of Medicine, University of Calgary, Calgary, AB Canada; 8 Department of Clinical Neurosciences, Cumming School of Medicine, University of Calgary, Calgary, AB Canada; 9 Centre for Health Informatics, Cumming School of Medicine, University of Calgary, Calgary, AB Canada; 10 Cumming School of Medicine, O’Brien Institute for Public Health, University of Calgary, Calgary, AB Canada; 11 Hotchkiss Brain Institute, Cumming School of Medicine, University of Calgary, Calgary, AB Canada; 12 Department of Medicine, Cumming School of Medicine, University of Calgary, Calgary, AB Canada; 13 Department of Radiology, Cumming School of Medicine, University of Calgary, Calgary, AB Canada; 14 Faculty of Nursing, Faculty of Medicine & School of Public Health, University of Alberta, Edmonton, AB Canada; 15 Department of Industrial Engineering, Dalhousie University, Nova Scotia, Canada; 16 Department of Community Health and Epidemiology, Department of Medicine (Neurology), Dalhousie University, Nova Scotia, Canada; 17 Ted Rogers Centre for Heart Research, Toronto, ON Canada; 18 Department of Medical Imaging, University Health Network, University of Toronto, Toronto, ON Canada; 19 Department of Medicine, University of British Columbia, Vancouver, BC Canada; 20 Department of Medicine, Division of Neurology, McMaster University, and Population Health Research Institute, Hamilton, ON Canada

**Keywords:** Cardiovascular risk factors, Hypertension, Diabetes, Dyslipidemia, Atrial fibrillation, Medication adherence, Medication discontinuation, Healthcare delivery

## Abstract

**Background:**

Health system disruptions since onset of the COVID-19 pandemic may have adversely impacted adherence to medications for common cardiovascular risk factors.

**Methods:**

We examined adherence to and discontinuation of incident prescriptions for medications treating hypertension, dyslipidemia, diabetes, and atrial fibrillation in Ontario, Alberta, and Nova Scotia, Canada. We compared the recent period (April 1, 2020 through most recently available follow-up: September 30, 2021 for Ontario; March 31, 2021 for Alberta; and March 31, 2022 for Nova Scotia) to the baseline, pre-pandemic period (April 1, 2014 through March 31, 2019). In each province, people aged ≥66 years with a valid health number and corresponding incident prescription were included. For each medication class, adherence in the recent period, defined as ≥ 0.80 proportion-of-days-covered (PDC), was compared to the pre-pandemic period using modified Poisson regression with robust error variance, adjusted for patient characteristics. Similarly adjusted Cox proportional hazards models compared hazard of discontinuation over one year of follow-up between the two time periods.

**Results:**

In the recent period, PDC ranged from 48.9% for dyslipidemia medications in Alberta to 82.2% for anticoagulants in Nova Scotia. Adherence was not different between periods, with the following exceptions: higher adherence in the recent period for antihypertensives (adjusted risk ratios [aRR] 1.08, 95% CI 1.06–1.10) and dyslipidemics (aRR 1.07, 95% CI 1.04–1.09) in Nova Scotia, and for antihyperglycemics (aRR 1.10, 95% CI 1.08–1.14) and anticoagulants (1.15, 95% CI 1.12, 1.18) in Alberta. Adherence was lower in the recent period only for antihypertensives in Alberta (aRR 0.95, 95% CI 0.93, 0.97). One-year rates of discontinuation ranged from 20.9% for anticoagulants in the Alberta recent period to 56.7% for antihypertensives in the Ontario baseline period. The adjusted hazard of discontinuation was lower or unchanged in the recent period for all medication classes.

**Conclusions:**

Despite significant health system disruptions since 2020, recent adherence to incident cardiovascular prescriptions was similar or better than before and rates of medication discontinuation were lower. However, interventions are still needed to improve existing, suboptimal adherence.

**Supplementary Information:**

The online version contains supplementary material available at 10.1186/s12872-025-04492-3.

## Background

Cardiovascular and vascular diseases are among the leading causes of death and disability globally. Medications to treat vascular risk factors are among the most effective tools to reduce their risk [1]. Patient adherence to medical therapy for hypertension, hyperlipidemia, diabetes, and atrial fibrillation have been shown to improve quality of life, survival, and cardiovascular health outcomes [2, 3].

With onset of the COVID-19 pandemic in 2020, health systems were disrupted by both direct changes to healthcare delivery and by indirect effects of public health protections (e.g., travel restrictions), temporary limitations in medication refills to prevent stockpiling and shortages, and patient-reported fear of infection [4–6]. Implementation of early pandemic-related policies were associated with lower medication adherence for chronic conditions such as asthma and inflammatory bowel disease [7]. Early identification of reduced adherence to medications for vascular risk factors is critical in preventing long-term negative consequences on cardiovascular events [1]. Potential impact on routine medical care, particularly medical care for cardiovascular and vascular risk factors, is not known. If identified, decline in medication adherence or persistence may require investment of resources or policy changes to better support patient health; on the other hand, lack of signal of worse adherence suggests adaptations may have been effective.

Therefore, our objective was to test the hypothesis that there were changes in adherence to and discontinuation of incident prescriptions for medications to treat hypertension, dyslipidemia, diabetes, or atrial fibrillation changed compared to before onset of the COVID-19 pandemic in the three Canadian provinces of Ontario, Alberta, and Nova Scotia.

## Methods

### Study setting and population

To assess whether adherence or persistence to common cardiovascular medications changed after 2020 across multiple universal health care systems, we created cohorts of individuals with incident prescriptions for medications to treat hypertension, dyslipidemia, diabetes, or oral anticoagulation for patients with atrial fibrillation between April 1, 2014 and March 31, 2022 in Ontario, Alberta, and Nova Scotia using population-level, linked administrative datasets. Supplemental Table 1 lists data sources for each variable, which include population-level administrative databases maintained in each province; these databases include vital statistics, sociodemographic data, and health system data, including medication prescriptions, as well as outpatient and inpatient diagnosis and billing codes. Briefly, medication prescription data came from each province’s drug benefit programmes (administered by the provincial ministries of health), which cover drug expenses for all residents in Alberta and residents 65 years and older in Ontario and Nova Scotia, who are eligible for each province’s publicly funded health insurance plan.

Combined, the populations of Ontario, Alberta, and Nova Scotia account for 53% of the adult population of Canada; each province administers its own universal health care system, and data are not shared routinely between provinces, so cohorts for each medication class were also stratified by province. Inclusion criteria were: age 66–105 years; valid healthcare number (required for data linkage); at least 1 year of complete follow-up; and no prior history of hospitalization for stroke, acute myocardial infarction, or peripheral artery disease (look-back window to the 2002, when the International Classification of Diseases 10th edition (ICD-10) became available). We included adults aged 66 years and older because individuals 65 years and older are eligible for government funding of medication prescriptions in all three provinces, thus ensuring a minimum look-back window of one year of prescription records.

In each province, we constructed four separate cohorts consisting of incident users of medications to treat: hypertension, dyslipidemia, diabetes, and anticoagulation for atrial fibrillation. Definitions for each condition and corresponding medication classes are included in Supplemental Table 1. For each of the four cohorts, the index date was the date on which medication was first dispensed within the period. Incident users were defined as having no record of dispensed medication within the same class within the previous year (maximum look-back date for medications of April 1, 2013).

### Ethics approval and consent to participate

This study adhered to the Declaration of Helsinki for medical research involving human subjects. Need for written informed consent was waived in Ontario in accordance with legislation, and in Nova Scotia and Alberta via review by institutional review boards (IRBs). Use of Ontario’s data in this project was authorized via legislation; Sect. 45 of Ontario’s Personal Health Information Protection Act sets out confidentiality and safety requirements for use of administrative data for research without additional research ethics board/IRB approval. In accordance with this legislation, Ontario datasets were deterministically linked using unique encoded identifiers and analyzed at ICES (formerly the Institute for Clinical Evaluative Sciences); IRB approvals were obtained from the Nova Scotia Health Research Ethics Board (REB #1027801) and the University of Calgary Conjoint Health Research Ethics Board (REB22-0339), respectively.

### Exposure definitions

The primary exposure was time period. The *baseline* period was used as the referent time period and was defined as April 1, 2014 through March 31, 2019, to allow a minimum of one year follow-up for the baseline period, before major health care system disruptions associated with onset of the COVID-19 pandemic. We excluded April 1, 2019 to March 31, 2020 to provide a minimum of one year of follow-up for the baseline period, and to ensure everyone included had the same duration of follow-up, patients who died within 365 days were excluded. This also excluded several months of widespread, temporary disruptions to health care delivery that occurred early in the pandemic but were not reflective of longer-term health system changes and likely would have biased results away from the null. The *recent* period, defined by onset of widespread health system disruptions that began during the early phase of the COVID-19 pandemic, began in all provinces on April 1, 2020 and ended at the most recent available data, which was different for each province at the time of this study: September 30, 2021 for Ontario, March 31, 2021 for Alberta, and March 31, 2022 for Nova Scotia.

### Outcome definitions

The primary outcome was the one-year medication adherence, defined by convention as the proportion of days covered (PDC) ≥ 80% for medication classes used to treat each condition (i.e., hypertension, dyslipidemia, diabetes, and atrial fibrillation, respectively) among incident users. (see Supplemental Table 1 for details) [8]. The secondary outcome of time-to-medication discontinuation, or, non-persistence was defined as the number of days between the index date (date of first prescription dispensing, i.e., the index date) and interruption in prescription refills (i.e., 30 days or more after the date of expected prescription refill).

### Statistical analyses

All analyses are presented separately for each condition of interest (hypertension, dyslipidemia, diabetes, and atrial fibrillation) and province (Ontario, Alberta, and Nova Scotia). Descriptive statistics for patients with incident dispensation for medications to treat one of the conditions of interest were stratified by province and presented as median (interquartile range), or frequency and proportion, as appropriate.

Crude and adjusted analysis compared PDC ≥ 80% for the baseline period versus recent period. Because the outcome was not rare, an estimated odds ratio from a logistic regression model will over-estimate the relative risk [9]. Therefore, we used modified Poisson regression models that incorporated a robust variance estimator to estimate the relative risk [10]. Adjusted models included the following clinically relevant covariates, chosen a priori because they were associated with both the exposure and outcome while not being within the causal pathway between them: age (continuous), sex (male/female), Charlson comorbidity index (< 2 or missing), and polypharmacy (≥ 5 prescribed medications at the time of index date), and community size. Analyses for Ontario and Nova Scotia were also adjusted for neighborhood income quintile, but these data were not available in Alberta.

Comparison of time-to-first-medication discontinuation for the baseline period versus recent period was conducted using Cox proportional hazards models, using time period as a fixed binary covariate and the same adjusting covariates as above. In planned sensitivity analyses, we used a less strict definition of non-persistence, defined as the first interruption in prescription refills longer than 90 days from the date of the last period covered.

For this hypothesis-generating analysis, the p-value threshold was set at 0.05 for all analyses. Analyses were conducted using STATA v18.0 (StataCorp LLC, Texas, USA) and SAS v9.4 (SAS Institute, Inc, Cary, NC).

## Results

Baseline demographic characteristics for each cohort by province are reported in Table 1, and exclusions are reported in Supplemental Table 2. Median age for patients with incident prescriptions for antihypertensives, dyslipidemics, and antihyperglycemics ranged from 71 to 73 years, while patients started on anticoagulation for atrial fibrillation were slightly older, with median ages 73 to 78 years. Approximately half of patients with incident antihyperglycemics were female, while the proportion of female patients for other conditions ranged between 53% and 56%. Baseline characteristics for baseline and recent periods were overall similar within each province. Across provinces, the monthly average number of incident prescriptions was 1,925 for antihypertensives, 1,824 for dyslipidemics, 763 for antihyperglycemics, and 424 for anticoagulants for atrial fibrillation.

**Table 1:** Baseline characteristics, stratified by province, for people with an incident prescription for an antihypertensive medication, dyslipidemia medication, antihyperglycemic medication, and anticoagulation medication*

	Ontario			Alberta			Nova Scotia		
	Baseline Period	Recent Period	Stand diff	Baseline Period	Recent Period	Stand diff	Baseline Period	Recent Period	Stand diff
	*n (%) unless otherwise specified*								
**Individuals with an incident prescription for an antihypertensive medication**									
Population size, N	261,527	61,674		82,693	12,700		18,611	7025	
Age, median years (Q1-Q3)	72 (68–78)	72 (68–77)	0.072	73 (69–79)	72 (69–78)	0.088	71 (68–77)	71 (68–75)	0.132
Female	145,410 (55.6)	34,768 (56.4)	0.016	44,853 (54.2)	7053 (55.5)	0.026	10,361 (55.7)	3,803 (54.1)	0.031
Neighborhood income quintile									0.030
Highest	52,022 (19.9)	13,108 (21.3)	0.034	Not available	Not available		3273 (17.6)	1298 (18.5)	
Next to highest	49,781 (19.0)	12,233 (19.8)	0.020				3698 (19.9)	1348 (19.2)	
Middle	52,874 (20.2)	12,390 (20.1)	0.003				3741 (20.1)	1446 (20.6)	
Next to lowest	54,208 (20.7)	12,247 (19.9)	0.022				4133 (22.2)	1547 (22.0)	
Lowest	51,951 (19.9)	11,518 (18.7)	0.030				3707 (19.9)	1370 (19.5)	
Unknown	691 (0.3)	178 (0.3)	0.005				59 (0.3)	16 (0.2)	
Rural residence	7,864 (12.8)	2,204 (13.7)	0.027	20,018 (24.2)	2734 (21.5)	0.064	7912 (42.5)	2829 (40.3)	0.052
Long-term care in prior 6 months	4534 (1.7)	828 (1.3)	0.032	1244 (1.5)	71 (0.56)	0.094	507 (2.7)	100 (1.4)	0.091
Hospitalization < 6 months before index date	17,526 (6.7)	4157 (6.7)	0.002	12,240 (14.8)	1534 (12.1)	0.080	1911 (10.3)	664 (9.5)	0.028
Charlson comorbidity index < 2 or missing	244,274 (93.4)	58,073 (94.2)	0.031	71,114 (86.0)	11,496 (90.5)	0.141	11,273 (60.6)	4,518 (64.3)	0.077
Polypharmacy	42,913 (16.4)	10,157 (16.5)	0.002	16,663 (20.2)	2742 (21.6)	0.035	6816 (36.6)	2439 (34.7)	0.040
Any hospitalization during 1-year follow-up	39,022 (14.9)	7753 (12.6)	0.068	14,875 (18.0)	1763 (13.9)	0.112	2684 (14.4%)	849 (12.1%)	0.069
**Individuals with an incident prescription for a dyslipidemia medication**									
Population size, N	249,313	66,767		68,053	13,076		17,211	7304	
Age, median years (Q1-Q3)	71 (68–76)	71 (68–75)	0.089	72 (68–77)	72 (68–76)	0.075	71 (68–76)	70 (68–75)	0.117
Female	133,896 (53.7)	36,956 (55.4)	0.033	33,766 (49.6)	6932 (53.0)	0.068	9714 (56.4)	4028 (55.1)	0.026
Neighborhood income quintile									0.047
Highest	49,142 (19.7)	14,315 (21.4)	0.043	Not available	Not available		2875 (16.7)	1346 (18.4)	
Next to highest	47,769 (19.2)	13,465 (20.2)	0.025				3385 (19.7)	1430 (19.6)	
Middle	50,091 (20.1)	13,304 (19.9)	0.004				3560 (20.7)	1494 (20.5)	
Next to lowest	52,082 (20.9)	13,377 (20.0)	0.021				3853 (22.4)	1598 (21.9)	
Lowest	49,655 (19.9)	12,132 (18.2)	0.044				3491 (20.1)	1419 (19.4)	
Unknown	574 (0.2)	174 (0.3)	0.006				47 (0.3)	17 (0.2)	
Rural residence	27,834 (11.2)	8023 (12.0)	0.027	15,408 (22.6)	2776 (21.2)	0.034	7,122 (41.4)	3,015 (41.3)	0.012
Long-term care in prior 6 months	1,812 (0.7)	424 (0.6)	0.011	549 (0.8)	62 (0.5)	0.042	289 (1.7)	64 (0.9)	0.072
Hospitalization < 6 months before index date	13,685 (5.5)	3794 (5.7)	0.008	10,919 (16.0)	1713 (13.1)	0.084	1691 (9.8)	627 (8.6)	0.043
Charlson comorbidity index < 2 or missing	233,585 (93.7)	62,884 (94.2)	0.021	56,533 (83.1)	11,473 (87.7)	0.133	10,009 (58.2)	4,630 (63.4)	0.107
Polypharmacy	57,168 (22.9)	14,916 (22.3)	0.014	18,031 (26.5)	3677 (28.1)	0.036	7,580 (44.0)	2,861 (39.2)	0.099
Any hospitalization during 1-year follow-up	31,789 (12.8)	7037 (10.5)	0.069	11,926 (17.5)	1773 (13.6)	0.110	2,348 (13.6%)	794 (10.9%)	0.085
**Individuals with an incident prescription for an antihyperglycemic medication**									
Population size, N	102,955	31,080		28,616	4862		6195	2700	
Age, median years (Q1-Q3)	72 (68–77)	72 (68–77)	0.023	72 (68–77)	72 (68–77)	0.048	71 (68–76)	71 (68–75)	0.037
Female	52,141 (50.6)	15,632 (50.3)	0.007	13,471 (47.1)	2231 (45.9)	0.024	3105 (50.1)	1295 (48.0)	0.043
Neighborhood income quintile									0.068
Highest	17,431 (16.9)	5537 (17.8)	0.023	Not available	Not available		952 (15.4)	465 (17.2)	
Next to highest	19,052 (18.5)	6043 (19.4)	0.024				1236 (19.9)	515 (19.1)	
Middle	21,093 (20.5)	6267 (20.2)	0.008				1221 (19.7)	562 (20.8)	
Next to lowest	22,378 (21.7)	6701 (21.6)	0.004				1469 (23.7)	591 (21.9)	
Lowest	22,741 (22.1)	6453 (20.8)	0.032				1301 (21.0)	556 (20.6)	
Unknown	260 (0.3)	79 (0.3)	0				16 (0.3)	11 (0.4)	
Rural residence	11,199 (10.9)	3393 (10.9)	0.001	7083 (24.8)	1001 (20.6)	0.100	2672 (43.1)	1095 (40.6)	0.053
Long-term care in prior 6 months	2116 (2.1)	482 (1.6)	0.038	358 (1.3)	25 (0.5)	0.079	142 (2.3)	43 (1.6)	0.051
Hospitalization < 6 months before index date	5832 (5.7)	1948 (6.3)	0.025	2975 (10.4)	470 (9.7)	0.024	504 (8.1)	177 (6.6)	0.061
Charlson comorbidity index < 2 or missing	92,516 (89.9)	28,086 (90.4)	0.017	22,125 (77.3)	4063 (83.6)	0.158	2,546 (41.1)	1,167 (43.2)	0.043
Polypharmacy	39,342 (38.2)	12,997 (41.8)	0.074	10,778 (37.7)	1925 (39.6)	0.040			
Any hospitalization during 1-year follow-up	13,219 (12.8)	3462 (11.1)	0.052	4695 (16.4)	673 (13.8)	0.072	864 (13.9%)	314 (11.6%)	0.069
**Individuals with a diagnosis of atrial fibrillation and incident prescription for an anticoagulant medication**									
Population size, N	61,561	16,086		15,435	2622		1534	561	
Age, median years (Q1-Q3)	77 (71–84)	76 (71–83)	0.049	78 (72–84)	77 (71–84)	0.037	75 (70–81)	73 (69–79)	0.183
Female	33,690 (54.7)	8580 (53.3)	0.028	8083 (52.4)	1397 (53.3)	0.018	767 (50.0)	265 (47.2)	0.055
Neighborhood income quintile									0.102
Highest	12,475 (20.3)	3430 (21.3)	0.026	Not available	Not available		263 (17.1)	117 (20.9)	
Next to highest	11,193 (18.2)	3064 (19.0)	0.022				281 (18.3)	103 (18.4)	
Middle	12,167 (19.8)	3172 (19.7)	0.001				328 (21.4)	109 (19.4)	
Next to lowest	12,990 (21.1)	3236 (20.1)	0.024				350 (22.8)	117 (20.9)	
Lowest	12,565 (20.4)	3126 (19.4)	0.024				312 (20.3)	115 (20.5)	
Unknown	171 (0.3)	58 (0.4)	0.015						
Rural residence	7847 (12.7)	2200 (13.7)	0.027	4215 (27.3)	695 (26.5)	0.018	671 (43.7)	216 (38.5)	0.210
Long-term care in prior 6 months	1783 (2.9)	353 (2.2)	0.045	417 (2.7)	58 (2.2)	0.032	44 (2.9)	10 (1.8)	0.072
Hospitalization < 6 months before index date	14,343 (23.3)	3637 (22.6)	0.016	8636 (56.0)	1528 (58.3)	0.047	550 (35.9)	188 (33.5)	0.049
Charlson comorbidity index < 2 or missing	48,692 (79.1)	12,780 (79.4)	0.009	9472 (61.4)	1656 (63.2)	0.037	747 (48.7)	292 (52.1)	0.067
Polypharmacy	31,202 (50.7)	7838 (48.7)	0.039	7996 (51.8)	1365 (52.1)	0.005	776 (50.6)	280 (49.9)	0.013
Any hospitalization during 1-year follow-up	17,940 (29.1)	4372 (27.2)	0.044	5468 (35.4)	868 (33.1)	0.049	253 (16.5%)	72 (12.8%)	0.081

* Baseline period is April 1, 2014 to March 31, 2019. Recent period for Ontario is April 1, 2020 to September 30, 2021, for Alberta is April 1, 2020 to March 31, 2021, and for Nova Scotia April 1, 2020 to March 31, 2022. For age, standardized difference refers to the difference in mean values

### Medication adherence by condition and province

**Fig. 1:**
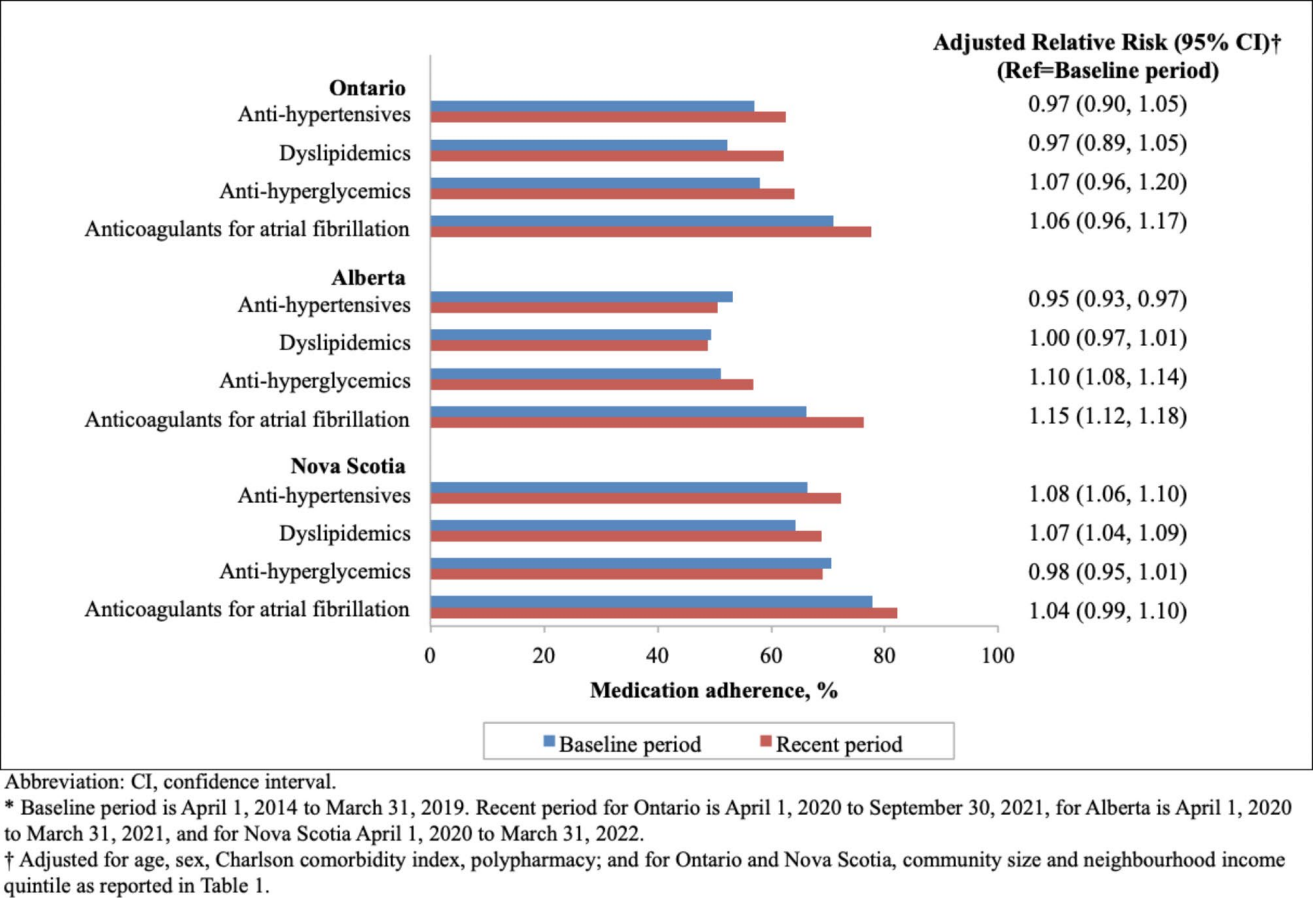
Medication adherence (proportion of days covered ≥ 80%) for people with an incident prescription for antihypertensive medication, dyslipidemia medication, antihyperglycemic medication, and anticoagulation medication*

PDC ≥ 80% in the baseline and recent periods (Fig. 1) varied by medication classes and provinces, ranging from as low as 48.9% (for dyslipidemic medications in the recent period in Alberta) to as high as 82.2% (for anticoagulants in the recent period in Nova Scotia). By medication classes and within provinces, PDC ≥ 80% was highest for oral anticoagulants and lowest for dyslipidemics.

**Fig. 2:**
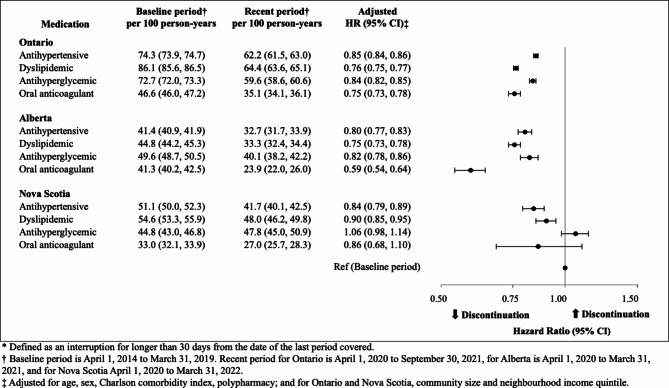
Medication discontinuation within one year of initiation*

Within medication classes, we did not detect differences in medication adherence for the recent period compared with the baseline period were found (Fig. 2, Supplemental Table 3), with the following exceptions: adjusted risk ratios (aRR) for adherence were higher in the recent period for antihypertensive and dyslipidemic medications in Nova Scotia (aRR 1.08 [95% CI 1.06–1.10] and 1.07 [95% CI 1.04–1.09], respectively), and for antihyperglycemics and anticoagulants in Alberta (aRR 1.10 [95% CI 1.08–1.14] and 1.15 [95% CI 1.12–1.18], respectively); adherence for antihypertensives was only lower in the recent period in Alberta (aRR 0.95 [95% CI 0.93–0.97]).

### Medication discontinuation by condition and province

Overall, medication discontinuation within one year occurred less often in the recent period compared to the baseline period (Fig. 2), ranging from as low as 20.9% (for anticoagulants in the recent period in Alberta) to as high as 56.7% (for antihypertensives in the baseline period in Ontario). Of the medication classes, discontinuation was least common for anticoagulants; otherwise, however, there was heterogeneity with regards to discontinuation across medication classes and provinces. The adjusted hazards of discontinuation over one year were lower in the recent period compared with the baseline period, with the exceptions of antihyperglycemics and oral anticoagulants in Nova Scotia, for which there was no detected difference in the hazard of discontinuation. In Ontario, adjusted hazard ratios (aHRs) for discontinuation in the recent period compared to the pre-pandemic period ranged from an aHR of 0.75 (95% CI 0.82–0.85) for antihyperglycemics to 0.85 (95% CI 0.84–0.86) for antihypertensives. In Alberta, aHR ranged from 0.59 (95% CI 0.54–0.64) for oral anticoagulants, to 0.82 (95% CI 0.78–0.86) for antihyperglycemics. In Nova Scotia, aHR for discontinuation in the recent period was 0.84 (95% CI 0.79–0.89) for antihypertensives, with aHR of 0.90 (95% CI 0.85–0.95) for discontinuation of dyslipidemics. Results were similar using a 90-day window to define discontinuation (Supplemental Table 4).

## Discussion

In this population-level study of incident prescriptions for medications to treat the common cardiovascular risk factors of hypertension, dyslipidemia, diabetes, and atrial fibrillation, we did not find evidence of recent declines in adherence. Despite widespread, long-term changes in healthcare delivery [4, 11–13], adherence to common cardiovascular medications was similar or better in the recent period. Furthermore, rates of medication discontinuation were also overall lower across conditions in the recent period compared with the baseline period, indicating that our findings did not reflect short-term stockpiling or other brief, temporary changes in adherence behaviors but instead may reflect a new or continued trend in medication adherence and persistence.

This work is unique in its focus on population-level adherence and persistence to incident prescriptions for common cardiovascular risk factors in universal health care systems managed by three different Canadian provinces. Our study adds to our understanding of recent trends in management of common chronic conditions such as lung disease, autoimmune conditions, which have generally identified a decline in adherence associated with onset of the COVID-19 pandemic [7, 14–16]. Our findings of similar or even improved cardiovascular medication adherence is a notable contrast that warrants further investigation. We cannot determine whether or to what degree sustained or improved adherence may have been related to the cardiovascular conditions, which can reasonably be managed via telemedicine and which was expanded in some locations as part of initial pandemic health system restructuring [17], or attributed to characteristics of the health systems or patients themselves. Identifying evidence of resilience despite significant health system disruptions and the worst global pandemic in a century may provide lessons to draw from and build on in future health system disruptions.

Rapid and widespread implementation of telemedicine may have offset fewer in-person visits while still allowing provision of high-quality medical care. Unlike other conditions that require complex care coordination or in-person interventions, response to treatment for hypertension, diabetes, dyslipidemia, and atrial fibrillation can be assessed by laboratory or home measurements, and medication changes can be performed remotely. Effectiveness of telemedicine programs varies by condition, implementation, patient population, and payment structure [4, 11, 18–24]. Rapid, widespread expansion of telemedicine may have lowered previous barriers to access such as patient travel, time off work, and arranging caregivers, but further investigation is required to fully understand the uptake and impact of telemedicine across patient populations.

Other factors may have also played a role in our findings. Cardiovascular risk factors may have been prioritized by both patients and clinicians, given public health messaging regarding their importance as risk factors for severe COVID-19 [25, 26]. Health system innovations, such as expanded pharmacist services including refilling chronic prescriptions for patients unable to access their family physician, providing vaccinations at local pharmacies may have supported medication adherence, and recent expansion of pharmacist scope-of-practice to include prescribing medications for low acuity conditions [12, 13]. Finally, limits on prescription durations during the first several months of the pandemic may have encouraged adherence by requiring more frequent and regular engagement with a healthcare professional, which is associated with better medication adherence, or otherwise changed patterns of medication adherence [27–29]. 

While it is reassuring that we did not find evidence of recent declines in medication adherence and in some cases found evidence that adherence improved slightly, it is important to note that improved medication adherence to common cardiovascular conditions is vital. Despite effective medical treatments, many patients continue to have uncontrolled but treatable cardiovascular risk factors because of suboptimal medication adherence. Renewed focus on prevention, early detection, and appropriate management of common cardiovascular risk factors is needed. Heart disease and stroke caused more deaths in 2021 than all forms of cancer and low respiratory tract infections, combined [30]. Previously declining or plateaued trends in incidence of cardiovascular risk factors and disease have stalled or even reversed in recent years [31, 32], and there is growing evidence that long-term effects of SARS-CoV-2 may further add to cardiovascular disease burden in coming decades [33]. ^,^ [34–37] While our analyses are overall reassuring and did not find overt evidence of deterioration in adherence for common cardiovascular medications, the overall adherence could be improved for all as PDC in the ranges of 50–80% in older adults at highest risk of cardiovascular disease are not optimal. Thus, continued investment of financial and human resources will be vital to ensure sustained success and potentially improve it over the coming years.

Health system changes and associations with changes in population-level health since 2020 have been complex and mixed. On the one hand, there is clear evidence of declines in access to primary care since 2020, with rising wait times for diagnostic imaging, surgeries, and speciality care and primary care [34–42] and evidence of barriers to access to care for asthma, inflammatory bowel disease, and seizure disorders [7, 43–45]. On the other hand, health systems rapidly deployed resources and restructured in early phases of the pandemic to ensure acute medical and surgical care [46, 47]. Recent increases is incident prescriptions for antihypertensive and antihyperglycemic medications may be explained by patient access to medical care for diabetes and hypertension despite health system disruptions, rising patient need, or both [31]. 

Further work is needed to fully identify barriers and facilitators of reliable, sustainable management of chronic cardiovascular risk factors in the face of health system disruptions and family physician shortages, including comparison across different types of health systems.

### Limitations

While we examined management of common vascular risk factors from several angles, it is possible that we did not detect other potential changes to the management of common cardiovascular conditions. Our findings apply to people who were able to access health care and may not generalize, for example, to people unable to access medical care or use telemedicine, people < 66 years of age, or to countries without universal healthcare. Further, we were unable to adjust for or explore potential heterogeneity across race or ethnicity because they are not currently measured consistently across Canada, nor were we able to adjust for socioeconomic status measured by neighborhood income quintile because that data were not available in Alberta. Future work should address possible effect modification by age, health system, and social determinants of health.

We cannot determine the cause for sustained or improved medication adherence with these available data. For example, sustained or improved adherence that occurred after onset of the COVID-19 pandemic may have been unrelated to the pandemic or resulting health policies. In the case of diabetes medications, rapid uptake of weekly dosed antihyperglycemic medications predated onset of the pandemic, patients with pre-diabetes of higher socioeconomic status may have had better access to clinicians and may have been more likely to start medical therapy than otherwise similar patients of lower socioeconomic status [48–50]. We did not include insulin as a category of anti-hyperglycemia medications. Adequate adherence was defined by convention as PDC ≥ 0.80, although this threshold may not reflect the disease control threshold for all conditions [8]. Finally, the longer-term influence of early pandemic healthcare disruptions are not known, so our findings may not generalize as health systems continue to evolve, including more recent changes to telemedicine compensation [1–3], worsening primary care access shortages, and reports of new barriers to access to chronic care including emergency department closures and increasing fragmentation of medical care [42, 51, 52].

## Conclusion

Patients prescribed new cardiovascular medications since 2020 were similarly or more adherent to medication compared with those who initiated these medications in 2014–2019, and they were less likely to discontinue these medications. Despite disruptions in healthcare in 2020–2021 and a growing shortage of family doctors, this suggests the population was able to access routine vascular preventive treatments during the study. However, future interventions are needed to address current, suboptimal medication adherence, as is ongoing monitoring to assess adherence trends over time.

## Electronic supplementary material

Below is the link to the electronic supplementary material.

Supplementary Material 1

## Data Availability

The datasets generalized and/or analysed during the current study are not publicly available due to privacy and security requirements, but portions may be available with institutional approval from the corresponding author on reasonable request.

## Abbreviations

aHR: Adjusted hazard ratio
aRR: Adjusted risk ratio
ICD-10: International Classification of Diseases 10th edition
PDC: Proportion days covered
