# Supplementary material for: Incident prescriptions for common cardiovascular medications: comparison of recent versus pre-2020 medication adherence and discontinuation in three universal health care systems
Source: BMC Cardiovasc Disord. 2025 Feb 5;25:82. doi: 10.1186/s12872-025-04492-3 (PMC11796216; doi:10.1186/s12872-025-04492-3)
Supplement: Supplementary file 1 — Supplementary Material 1 [file 12872_2025_4492_MOESM1_ESM.docx]

**Supplemental materials**

**Supplemental Table 1:** Administrative data sources and variable definitions

**Supplemental Table 2:** Cohort creation charts

**Supplemental Table 3**: Medication adherence, stratified by province, for people with an incident prescription for an antihypertensive medication, dyslipidemia medication, antihyperglycemic medication, and anticoagulation medication

**Supplemental Table 4:** Medication discontinuation within one year of initiation using a 90-day look forward window

| **Supplemental Table 1: Administrative data sources and variable definitions*** | | | |
| --- | --- | --- | --- |
| **Variable** | **Province** | **Database** (see Glossary below table for database descriptions) | **Definition** |
| Age | Ontario | Registered Person Database | Age at index date |
|  | Alberta | Pharmacy Information Network |  |
|  | Nova Scotia | Insured Patient Registry MASTER |  |
| Sex | Ontario | Registered Person Database | Male, Female |
|  | Alberta | Pharmacy Information Network |  |
|  | Nova Scotia | Insured Patient Registry MASTER |  |
| Ischemic stroke | All | Canadian Institute for Health Information Discharge Abstract Database | ICD-9: 434, 436, 362.3  ICD-10: I63 (excluding I63.6), I64, H341 |
| Intracerebral haemorrhage | All | Canadian Institute for Health Information Discharge Abstract Database | ICD-9: 430, 431  ICD-10-CA: I60, I61 |
| Coronary artery disease | All | Canadian Institute for Health Information Discharge Abstract Database | ICD-9: 410  ICD-10: I21, I22  CCP: 4802, 4803, 481  CCI: 1IJ60, 1IJ57GQ, 1IJ54, 1IJ76 |
| Peripheral artery disease | All | Canadian Institute for Health Information Discharge Abstract Database | ICD-9: 4402, 4413, 4414, 4439, 4442 ICD-10-CA: I702, I713, I714, I739, I743, I744  CCP: 5012  CCI: 1JE57, 1JE60, 1JE87 |
| Antihypertensive medications | Ontario | Ontario Drug Benefit | ACE inhibitors, ARBs, beta blockers, calcium channel blockers, centrally acting anti-adrenergics |
|  | Alberta | Pharmacy Information Network |  |
|  | Nova Scotia | Seniors Pharmacare |  |
| Dyslipidemia medications | Ontario | Ontario Drug Benefit | Anion exchange resins, calcium blockers antilipemic combinations, ezetimibe, fibrates, niacinamide, statins and other antilipemic medications |
|  | Alberta | Pharmacy Information Network |  |
|  | Nova Scotia | Seniors Pharmacare |  |
| Antihyperglycemic medications | Ontario | Ontario Drug Benefit | Oral antihyperglycemics |
|  | Alberta | Pharmacy Information Network |  |
|  | Nova Scotia | Seniors Pharmacare |  |
| Oral anticoagulants for atrial fibrillation | Ontario | Canadian Institute for Health Information Discharge Abstract Database, NACRS, Ontario Health Insurance Plan, Ontario Drug Benefit | Atrial fibrillation definition: (1 hospitalization or 1 ED visit with ICD-10-CA I48) or (4 physician claims with diagnosis code 427 within 1 year) in the 5 years prior to index date  Medication classes: anticoagulants, vitamin K antagonists |
|  | Alberta | Pharmacy Information Network |  |
|  | Nova Scotia | Seniors Pharmacare, Canadian Institute for Health Information Discharge Abstract, National Ambulatory Care Reporting System, MED Physicians Billing |  |
| Polypharmacy | Ontario | Ontario Drug Benefit | ≥5 dispensed medications of unique medication subclass in the 90 days prior to index date |
|  | Alberta | Pharmacy Information Network |  |
|  | Nova Scotia | Seniors Pharmacare |  |
| Proportion days covered | All | see above | % of days with medication coverage, censored at 1-year or date of death  For patients hospitalized during follow-up, PDC calculation assumed medication was continued during hospitalization (days in hospital were counted in the denominator) |
| **Abbreviations:** ACE indicates angiotensin converting enzyme; ARB, angiotensin receptor blocker; ED, emergency department visit; ICD, International Classification of Diseases  * 5-year look-back window was used, unless otherwise stated.  **Database descriptions:**  CIHI DAD – Contains individual-level administrative, clinical and demographic data from the discharge abstracts of all acute care hospitals in Canada (except Quebec), including admission and discharge dates, diagnoses, comorbidities and procedures.  MASTER, RPDB – Provide basic demographic information about anyone who has ever been registered for Nova Scotia and Ontario’s tax-funded health insurance plans, respectively, e.g., date of birth, date of death, sex, residential address and time periods of eligibility.  MED Physicians Billing, OHIP – Captures all reimbursement claims made by registered health care providers who are eligible to claim under Nova Scotia and Ontario’s health insurance plans, respectively, and includes information on service dates, diagnoses and services provided.  NACRS – Contains individual-level data about institution-based ambulatory care in Canada, specifically care provided in EDs, out-patient clinics, and day surgeries.  ODB, Pharmacy Information Network and Seniors Pharmacare – Contains information about prescription drug claims covered by and made to Ontario, Alberta and Nova Scotia’s drug benefit programmes, respectively, e.g., dispensing date, drug identifier and quantity supplied. | | | |

| **Supplemental Table 2: Cohort creation charts** | | | | |
| --- | --- | --- | --- | --- |
| **ONTARIO** | **Antihypertensives** | **Dyslipidemics** | **Antihyperglycemics** | **Anticoagulants** |
| **At least 1 claim between 2014.04 and 2022.03, and no claim in year prior to first claim during period** | **472,644** | **482,125** | **222,089** | **410,655** |
| Exclude non-Ontario residents or if age <66 or >105 on index date | 935 | 757 | 331 | 182 |
| Exclude if prior hospitalization for stroke, coronary artery disease, peripheral artery disease | 53,878 | 81,307 | 44,690 | 103,375 |
| Exclude if no history of AF diagnosis in prior 5 years | N/A | N/A | N/A | 200,816 |
| Exclude if died within 365 days after index date** | 17,097 | 7814 | 6843 | 10,756 |
| Exclude if index date between 2019.04 and 2020.03 | 50,098 | 46,358 | 21,641 | 11,888 |
| Exclude if index date >2021.09 due to availability of follow-up data | 27,435 | 29,809 | 14,549 | 5991 |
| **Final cohort included** | **323,201** | **316,080** | **134,035** | **77,647** |
|  |  |  |  |  |
| **ALBERTA** | **Antihypertensives** | **Dyslipidemics** | **Antihyperglycemics** | **Anticoagulants** |
| **At least 1 claim between 2014.04 and 2022.03, and no claim in year prior to first claim during period*** | **128,912** | **115,606** | **48,086** | **26950** |
| Exclude if age <66 or >105 year on index date, or if died within 365 days after index date | 5,272 | 3019 | 1580 | 2887 |
| Exclude if index date between 2019.04 and 2020.03 | 15,112 | 15,242 | 5734 | 3038 |
| Exclude if index date >2021.03 due to availability of follow-up data | 13,140 | 16,218 | 7296 | 2969 |
| **Final cohort included** | **95,393** | **81,129** | **33,478** | **18,057** |
|  |  |  |  |  |
| **NOVA SCOTIA** | **Antihypertensives** | **Dyslipidemics** | **Antihyperglycemics** | **Anticoagulants** |
| **At least 1 claim between 2014.04 and 2022.03, and no claim in year prior to first claim during period** | **65,803** | **62,643** | **23,601** | **55,233** |
| Exclude if no history of AF diagnosis in 5 years before or 1 year after index date | N/A | N/A | N/A | 50,495 |
| Exclude if invalid or unknown birth date or sex, or invalid death date | 22 | 23 | 11 | 1597† |
| Exclude if age <66 or > 105 years on index date | 29,955 | 27,952 | 10,482 |  |
| Exclude if prior hospitalization for stroke, coronary artery disease, peripheral artery disease | 4992 | 5824 | 2468 | 592 |
| Exclude if < 365 days of follow-up from index date | 1520 | 917 | 417 | 202 |
| Exclude if index date between 2019.04 and 2020.03 | 3678 | 3412 | 1328 | 252 |
| **Final cohort included** | **25,636** | **24,515** | **8895** | **2095** |
| * Patients with a history of hospitalization for stroke, coronary artery disease or peripheral artery disease were excluded prior to cohort creation.  † Steps combined due to n < 6 for exclusion of invalid or invalid or unknown birth date or sex, or invalid death date.  ** Individuals who died within 365 days of the index date were excluded to ensure the same duration of follow-up and because prescriptions are frequently changed in the year prior to death and may reflect intention de-prescribing rather than non-adherence or non-persistence[1] | | | | |

**Reference:**

1. Johansson KS, Petersen TS, Christensen MB, Pottegard A: **Methodological Considerations for Describing Medication Changes in Relation to Clinical Events and Death: An Applied Example in Patients with Type 2 Diabetes and Cancer**. *Drugs Aging* 2023, **40**(11):1009-1015.

| **Supplemental Table 3: Medication adherence, stratified by province, for people with an incident prescription for an antihypertensive medication, dyslipidemia medication, antihyperglycemic medication, and anticoagulation medication*** | | | | | | | | | | | | |
| --- | --- | --- | --- | --- | --- | --- | --- | --- | --- | --- | --- | --- |
| **Medication class** | **Ontario** | | | | **Alberta** | | | | **Nova Scotia** | | | |
| **Proportion of days covered ≥ 80%** | **Baseline**  **period**  **n (%)** | **Recent period**  **n (%)** | **RR (95% CI)**  **(Ref=baseline period)** | | **Baseline**  **period**  **n (%)** | **Recent period**  **n (%)** | **RR (95% CI)**  **(Ref=baseline period)** | | **Baseline**  **period**  **n (%)** | **Recent period**  **n (%)** | **RR (95% CI)**  **(Ref=baseline period)** | |
|  |  |  | **Crude** | **Adjusted†** |  |  | **Crude** | **Adjusted†** |  |  | **Crude** | **Adjusted†** |
| Anti-  hypertensive | 149,279 (57.1) | 38,530 (62.5) | 1.09  (1.09, 1.10) | 0.97  (0.90, 1.05) | 44,023 (53.2) | 6,420 (50.6) | 0.95  (0.93, 0.97) | 0.95  (0.93, 0.97) | 12,346 (66.3) | 5077 (72.3) | 1.09  (1.07, 1.11) | 1.08  (1.06, 1.10) |
| Dyslipidemic | 130,247 (52.2) | 41,434 (62.1) | 1.19  (1.18, 1.20) | 0.97  (0.89, 1.05) | 33,710 (49.5) | 6,388 (48.9) | 1.00  (0.97, 1.01) | 1.00  (0.97, 1.01) | 11,066 (64.3) | 5027 (68.8) | 1.07  (1.05, 1.09) | 1.07  (1.04, 1.09) |
| Anti-  hyperglycemic | 59,673 (58.0) | 19,903 (64.0) | 1.10  (1.09, 1.12) | 1.07   (0.96, 1.20) | 14,653 (51.2) | 2,761 (56.8) | 1.11  (1.08, 1.14) | 1.10  (1.08, 1.14) | 4375 (70.6) | 1866 (69.1) | 0.98  (0.95, 1.01) | 0.98  (0.95, 1.01) |
| Anticoagulant for atrial fibrillation | 43,723 (71.0) | 12,498 (77.7) | 1.09  (1.08, 1.10) | 1.06  (0.96, 1.17) | 10,210 (66.2) | 1,999 (76.2) | 1.15  (1.13, 1.18) | 1.15  (1.12, 1.18) | 1195 (77.9) | 461 (82.2) | 1.05  (1.01, 1.11) | 1.04  (0.99, 1.10) |
| Abbreviations: CI indicates confidence interval; and RR, relative risk.  * Baseline period is April 1, 2014 to March 31, 2019. Recent period for Ontario is April 1, 2020 to September 30, 2021, for Alberta is April 1, 2020 to March 31, 2021, and for Nova Scotia April 1, 2020 to March 31, 2022.  † Adjusted for age, sex, Charlson comorbidity index, and polypharmacy; and for Ontario and Nova Scotia, community size and neighborhood income quintile as reported in Table 1. | | | | | | | | | | | | |

| **Supplemental Table 4. Medication discontinuation within one year of initiation using a 90 day look forward period** | | | | | | |
| --- | --- | --- | --- | --- | --- | --- |
|  | **Ontario** | | **Alberta** | | **Nova Scotia** | |
| **Medication discontinuation (90 day look forward)** | **Baseline period,**  **per 100 person- years (95% CI)** | **Recent period,**  **per 100 person-years (95% CI)** | **Baseline period,**  **per 100 person-years (95% CI)** | **Recent period,**  **per 100 person-years (95% CI)** | **Baseline period,**  **per 100 person-years (95% CI)** | **Recent period,**  **per 100 person-years (95% CI)** |
| Antihypertensive | 47.8 (47.5, 48.1) | 41.7 (41.2, 42.3) | 17.1 (16.8, 17.4) | 13.9 (13.2, 14.6) | 32.6 (31.7, 33.5) | 25.4 (24.1, 26.7) |
| Dyslipidemic | 52.6 (52.2, 52.9) | 39.7 (39.2, 40.3) | 18.3 (18.0, 18.7) | 13.7 (13.1, 14.4) | 33.0 (32.1, 33.9) | 27.0 (25.7, 28.3) |
| Antihyperglycemic | 40.7 (40.3, 41.2) | 35.7 (35.0, 36.4) | 19.2 (18.7, 19.7) | 14.9 (13.8, 16.1) | 22.5 (21.3, 23.8) | 23.1 (21.2, 25.1) |
| Oral anticoagulation for atrial fibrillation | 25.5 (25.1, 25.9) | 20.6 (19.9, 21.4) | 11.2 (10.7, 11.8) | 7.0 (6.0, 8.1) | 17.8 (15.7, 20.2) | 13.6 (10.7, 17.1) |

Abbreviation: CI indicates confidence interval.
